# An integrative review of defining characteristic of the nursing diagnosis “spiritual distress”

**DOI:** 10.1002/nop2.1574

**Published:** 2023-01-10

**Authors:** Fateme Eshghi, Lida Nikfarid, Armin Zareiyan

**Affiliations:** ^1^ Student Research Committee, School of Nursing and Midwifery Shahid Beheshti University of Medical Sciences Tehran Iran; ^2^ Department of Pediatric Nursing, School of Nursing and Midwifery Shahid Beheshti University of Medical Sciences Tehran Iran; ^3^ Public Health Department, Health in Disaster & Emergencies Department, Nursing Faculty Aja University of Medical Sciences Tehran Iran

**Keywords:** nursing diagnosis, spiritual distress, spirituality

## Abstract

**Aim:**

The aim of this study was to identify the defining characteristics of spiritual distress (00066).

**Design:**

This study was conducted by integrated review method using Broom method.

**Methods:**

PubMed, ProQuest, Web of Science, Embase, Scopus, and Cochran Library, and Persian scientific databases were searched from January 2010 to December 2020.

**Results:**

Twenty‐one article and 74 defining characteristics were identified. 33 of these defining characteristics was mentioned in NANDA. The criteria with the highest frequency and repetition in articles were lack of peace, lack of hope, change in anger behaviour, lack of meaning in life, change in fear and crying behaviour, Concern about belief and values system and/or God.

**Conclusion:**

Some of the spiritual distress defining characteristics overlap with other nursing diagnoses, including anxiety and hopelessness. Clinical and content validation studies need to be conducted and the main criteria for diagnosing spiritual distress in different cultures and religions need to be identified.

## 1 INTRODUCTION

In recent decades, health‐related disciplines have paid remarkable attention to the issue of spirituality, as evidenced by the growing trend of publishing research and theoretical articles on the subject of spirituality and related concepts (Bornet et al., 2016). In line with the reported positive impact of spirituality on health, other reports have focused on the relationship between spirituality and the negative consequences of illness and hardships of life (de Oliveira Maraldi, 2020; Monod et al., 2010; Smucker, 1996; Tanyi, 2002). According to evidence, some people have feelings such as being punished, being purified, and guilty in the experiences like disasters and crises (Sipon et al., 2015; Tanyi, 2002). According to researches neglecting the patient's spiritual needs can lead to feelings of isolation and spiritual distress (Narayanasamy, 2006). North American Nursing Diagnosis Association‐International (NANDA‐I) introduced spiritual distress (00066) as a nursing diagnosis in 1978. Then in the last version (2018–2020), spiritual distress was defined as a state of suffering related to impaired ability to experience meaning‐giving in life, through connection to self, others, the world and nature, and a superior being (Ackley et al., 2021). The NANDA‐I describes responses of persons to identified health problems as *the defining characteristics* of nursing diagnoses (Assis et al., 2018; Carpenito‐Moyet, 2006). The defining characteristics are a format to facilitate the description of signs and symptoms of a response to an identified health problem which helps nurses diagnose what they need to intervene (Assis et al., 2018; Carpenito‐Moyet, 2006). Without enough evidence to support the validity of nursing diagnoses, nurses may find the defining characteristics non‐representative and uncertain. The diagnoses will not be confident if their content and clinical validity remain un‐investigated (Caldeira et al., 2017a; Romeiro et al., 2017). Researchers suggest for nursing diagnoses validation, one should not be satisfied with its defining characteristics in NANDA, and all defining characteristics should be obtained through review studies or concept analysis and included in the validation process. Also emphasize the importance of literature review as initial step for nursing diagnosis validation (Creason, 2004; Fehring, 1987). An integrated review is a specific review method to provide a more comprehensive understanding of a particular phenomenon or health care problem. This type of review summarizes past empirical or theoretical literature. Subsequently this method play a larger role in evidence‐based practice initiatives and better illustrates the inherent complexity of all nursing issues (Whittemore & Knafl, 2005).

### 1.1 Background

Religious and spiritual values and attitudes of people are challenged in times of illness and loss. These beliefs include their meaning given to life, sense of hope, and faith. Consequently, the person may experience a sense of doubt, confusion, and tension (Kord & Biadar, 2019; Koslander et al., 2021; Mrdjenovich, 2019). Concepts such as religious struggle, spiritual stress, and religious anguish thus found their way into health care providers' literature. Spiritual distress happens when persons are at risk of experiencing disruption in their value and belief system, which are the cause of hope, strength, and the meaning of life for them. In this case, the persons may need specialized care considerations provided by healthcare providers to prevent negative consequences such as reduced quality of life, less personal satisfaction, and wellness (Hatamipour et al., 2018; Narayanasamy, 2006). Thus, spiritual distress was included in nursing diagnoses as a human response to health and illness experiences. Nursing diagnosis is the second step; and an essential component of the professional nursing process. Nursing diagnoses as the patient's responses to health problems; explain the patient's unique experience. They are considered as the core concepts of nursing care (Herdman & Kamitsuru, 2017). To investigate the reliability and validity of proposed nursing diagnoses, it is essential to frequently redefine the components of every nursing diagnosis, including the defining characteristics, risk factors, and labels in different settings and populations. The NANDA‐I emphasizes the necessity of conducting studies that measure the validity of the nursing diagnoses (Caldeira et al., 2017b; Karaca & Aslan, 2018). A valid nursing diagnosis is evidence‐based and can resist criticism from professional nurses. Validation adds to their clinical value and makes them more confidently used by nurses in clinical practice, nursing education, and nursing research (Fehring, 1987; Romeiro et al., 2017). In 1987, Fehring published an article to propose a systematic process for the validation of nursing diagnoses (Fehring, 1987). The method was used in some studies aimed the validation of spiritual distress (Caldeira et al., 2013; Caldeira et al., 2016; Caldeira et al., 2017a, 2017b; Chaves, Carvalho, Terra, & Souza, 2010; Pehler, 1997). The dependence of culture and religion on spirituality is one of the main reasons for the numerous studies done to validate the diagnosis of spiritual distress nursing (Erickson & Carlson, 2014). Research has recently looked at the prevalence of spiritual distress and emphasized prioritizing it. One of the responsibilities of nurses is to pay attention to the spiritual aspects of care and create a therapeutic environment for patients. As part of comprehensive care, healthcare providers must acquire the skills needed to identify patients' spiritual needs and provide care beyond their physical needs (Hatamipour et al., 2015). Considering that spiritual distress is a complex, abstract and multidimensional phenomenon influenced by the cultural and religious context of the individual, determining the criteria that accurately define spiritual distress is an important issue. Nursing care should be in line with culture and religion. This integrated review was designed to determine the defining characteristics of spiritual distress as the first step of a validation study, as the integrated review demonstrates a more comprehensive understanding and knowledge of the phenomenon.

### 1.2 Research question

The research question was “What are the defining characteristics of spiritual distress in the literature which are precise and valid to include into the list of its defining characteristics in the NANDA‐I list?” As a preliminary step, the researcher tried to achieve a clear philosophical‐theoretical insight into the subject and find an accurate and precise method for reviewing resources.

## 2 THE REVIEW

### 2.1 Design

This integrative review used a method proposed by Broom to provide a comprehensive methodological review, including every type of research for creates the existing body of knowledge about the topic and evaluates its validity for application in clinical practice (Broome & Company, 2000).

### 2.2 Method

The steps of Broom methodology of integrative review are: (1) Problem identification (2) Literature search (3) Data evaluation (4) Data analysis, which includes data reduction, data display (data display), data comparison, final drawing and verification (synthesis) (5) Findings presentation (Broome & Company, 2000).

### 2.3 Search methods

The researcher conducted a preliminary search on Google Scholar to find an insight into the subject and identify keywords for the consequent search. Medical Subject Headings‐MESH has not included spiritual distress in its list of headings. So, access to the target resources was difficult. The researcher determined the alternative terms for spiritual distress in MESH. Then, PubMed, ProQuest, Web of Science, Embase, Scopus, and Cochran Library, and Persian scientific databases were searched, considering between the time of January 2010 and December 2020. Search terms included following key words/MESH terms and their synonyms: spirituality, spiritual distress and nursing diagnosis. Terms were searched alone and combined with Boolean Operators (and/or). Search syntax used in PubMed was:((Spiritualities[tiab] OR Spirituality[tiab] OR religion[tiab] OR “Spiritual anguish”[tiab] OR “Spiritual distress”[tiab]) AND (“Diagnosis Nursing”[tiab] OR “Diagnoses Nursing”[tiab] OR “Nursing Diagnoses”[tiab] OR “Nursing diagnosis”[tiab])).

#### 2.3.1 Inclusion and exclusion criteria

The included resources were the report of studies in English and Persian language that explicitly described spiritual distress or related subjects, accessible as full‐texts, consisting of the specifics of spiritual distress to adopt them as nursing diagnoses. Excluded resources were articles that did not provide information about defining characteristics of spiritual distress and unpublished or pre‐publication stage manuscripts.

The search included all various designs of study. Eligible resources were selected based on the relevance of their titles and abstracts. Then, full‐text versions of the determined articles were obtained. These articles also were evaluated by the researchers in terms of their eligibility. The researchers checked the reference lists of selected articles to find any potential additional articles.

#### 2.3.2 Search outcomes

A total of 283 resources were obtained in the initial search, all of which were in English. The search resulted in 23 resources from PubMed, 24 resources from Embase, 126 resources from Web of Science, 28 resources from Scopus, 71 published texts from ProQuest, one resource from Cochrane Library, and ten resources from other sources. To improve the accuracy of search results, the researcher searched the texts with an interval of 2 days again, and the same results were obtained. After excluding duplicates, the remaining 160 articles were screened based on their title and abstract, and in the next step were evaluated.

#### 2.3.3 Data evaluation

The evaluation of resources was challenging. Spirituality and spiritual distress have a wide area of related literature. So, the researchers considered only resources guiding them to find more features of spiritual distress. Many of the obtained resources focused on examining the relationship between spiritual distress and other variables, developing instruments, or explaining spiritual distress as a component of spiritual health‐related knowledge. From Whittemore & Knafl's point of view, there is no standard criterion for evaluating and interpreting the quality of the texts obtained in the search, and this is highly dependent on the sampling framework. Since the resources had various designs, scoring their quality was not possible (Whittemore & Knafl, 2005). Therefore, to evaluate them, the researchers used a method given from the field of historical studies. The research studies were evaluated based on their strength, originality, representativeness, and relevance to the subject of the study. Therefore, the resources were coded based on two criteria related to this review: 1‐ Theoretical and methodological rigour 2‐ Relevance of the data in a two‐point table (high and low). No resource was excluded based on this scoring system. But resources with less rigour and relevance involved less in the analysis process. Theoretical resources were also evaluated based on the thematic relevance and accuracy presented in the report.

#### 2.3.4 Data reduction, display, and comparison

This review is reported according to the Preferred Reporting Items for Systematic Reviews and Meta‐Analyses(Moher et al., 2009). The first step in reducing data is establishing a general information classification system to manage data obtained from various methodologies. The researchers used a pre‐determined data‐reduction sheet. The classification of information separated nursing from non‐nursing articles; and articles that describe spirituality in general from spiritual health and distress. Then the data obtained from the 160 articles were summarized and organized to deduce which ones could help extract the defining characteristics of spiritual distress.

Along with reducing the number of articles, the resources were constantly being moved between subclasses determined in the previous step. To assign each resource to the classes, all the members of the research team reached a consensus. The articles with overlapping contents were clustered by researchers, checked by the third researcher. We selected the resources if they were helpful for extraction of the characteristics of spiritual distress such a way a nurse can determine this health problem, were being chosen. Finally, 28 articles were included in the synthesis phase.

#### 2.3.5 Final drawing and verification (synthesis)

The process of summarizing and classifying the data of submitted articles into this phase enabled the researchers to manage them based on the research question. The researchers read the articles. Ten articles were excluded after reading the full text due to their lack of information that can be used to develop the defining characteristics of spiritual distress. The researchers checked the reference lists of selected articles to find any potential additional articles. Three articles were added. To ensure the accuracy of the synthesis, the researcher performed the analysis and reduction of resources with an interval of 2 days. Once again, the same results were obtained. In the end, 21 articles were designated to spirituality dimensions described by most of the theories and conceptualization related to this topic. The features of spiritual distress were coded and assigned to one of the four dimensions of God, nature, others, and self. A third researcher checked the codes. In the case of overlapping codes (features of spiritual distress), the one that best represents spiritual distress was chosen for the final overview.

## 3 RESULTS

### 3.1 Descriptive findings

**FIGURE 1:**
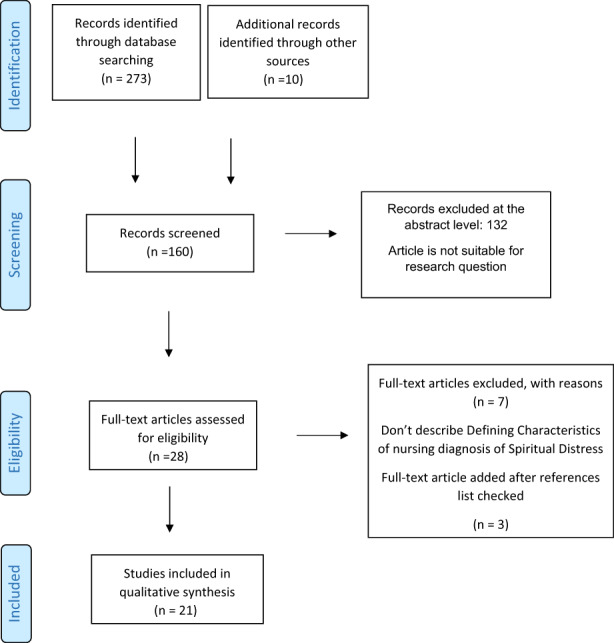
PRISMA flow chart for study selection

From 283 empirical and theoretical literature identified, 21 articles were included in the review. PRISMA‐flow chart and article selection show in the Figure 1. The majority of resources were developed in the field of nursing *N* = 15 (71.42%) followed by the medicine *N* = 4 (19.04%). Only two of the resources (9.54%) were developed in the other healthcare disciplines.

Most of the resources belonged to the years between 2016–2020. Methodologically, 5 articles were related to the validation of the nursing diagnosis “spiritual distress”, one of which was an evaluation of content validity and the rest were clinical validation. The methodology of the other articles were 2 integrated reviews, 3 tool development, 4 cross‐sectional descriptive, 1 cross‐sectional‐exploratory, 1 review, 1 Meta‐synthesis, 2 qualitative, 1 action research (patient education), and 1 spiritual care. The studied populations were patients with cancer (42.85%), the elderly (9.52%), people with chronic kidney disease (4.76%), people with chronic disease (4.76%), people with AIDS (4.76%), and patients with schizophrenia (4.76%) (Table 1).

**TABLE 1:** The characteristics of reviewed article in the literature review

Ref code for table 2	Authors name (year of publication (ref))	Methodology	Study sample	Authors	Study place	Study focus
1	Chaves, Carvalho, Terra, & Souza, 2010	Diagnostic content validation	Nurses	Nurses	Brazil	Defining characteristics of spiritual distress
2	Chaves, Carvalho, & Hass, 2010	Diagnostic clinical validation	Patients with chronic renal disease	Nurses	Brazil	Defining characteristics of spiritual distress
3	Caldeira et al., 2013	Integrative literature review	–	Nurses	Portugal	Defining characteristics of spiritual distress
4	Caldeira et al., 2014	Clinical validation	Elderly patients with cancer	Nurses	Portugal	Factors of the spiritual distress diagnosis.
5	Hatamipour et al., 2015	Qualitative approach	Patients with cancer	Nurses	Iran	Spiritual needs
6	Caldeira et al., 2016	Diagnostic clinical validation	Patients with cancer	Nurses	Portugal	Defining characteristics of spiritual distress
7	Caldeira et al., 2017a	Diagnostic clinical validation	Patients with cancer	Nurses	Portugal	Defining characteristics of spiritual distress
8	Pinho et al., 2017	Cross‐sectional study	People living with HIV/AIDS	Nurses	Brazil	Impaired religiosity and Spiritual distress nursing diagnosis
9	Romeiro et al., 2017	Discussion about spirituality	Infertile couples	Nurses	Portugal	Spirituality and the assessment of spiritual needs
10	Schultz et al., 2018	Cross‐sectional study	Patients with cancer	Physicians	Israel	Cultural expression and key indicators of spiritual distress
11	Martins & Caldeira, 2018	Synthesis of qualitative studies	Patients with cancer	Nurses	Portugal	Experience of spiritual distress as lived
12	Glenn & Pieper, 2019	Review	–	Nurses	USA	Spirituality, spiritual distress, and forgiveness
13	Silva et al., 2019	Cross‐sectional study	Patients with cancer	–	Brazil	Relation between spiritual distress and Religious/spiritual coping
14	Institute for Innovation in Palliative Care, 2016	Patient education series	–	Community Care of Brooklyn and MJHS Institute for Innovation in Palliative Care	USA	Spiritual distress
15	Velosa et al., 2017	Cross‐sectional study	Palliative care patients	Nurses	Portugal	Clinical indicators of depression and spiritual distress
16	Monod et al., 2010	Scale developing	Older hospitalized patients	Physicians	Switzerland	Spiritual distress
17	Monod et al., 2012	Validation tool	Older hospitalized patients	Physicians	Switzerland	Spiritual distress
18	Bhatnagar et al., 2017	Cross‐sectional study	Patients with cancer	Physicians	India	Signs of spiritual distress
19	Ku et al., 2010	Validation tool	Patients with cancer	Nurses	Taiwan	Spiritual distress
20	Simão et al., 2015	Integrative literature review	–	Nurses	Brazil	Defining characteristics of spiritual distress
21	Yang et al., 2012	Qualitative approach	Patients with schizophrenia	Nurses	Taiwan	Spirituality

### 3.2 Coding

A total of 244 initial codes were extracted from the detailed review of articles. After excluding duplicate codes and continuous comparisons as a method of data analysis, 74 criteria that could help describe the defining characteristics of spiritual distress were finally deduced, including a wide range of related psychological, physical, and social health‐related symptoms. By comparing and moving the codes, the main category called spiritual distress, and its four main subcategories include 1‐ Spiritual suffering connection to self (46 codes) 2‐ Spiritual suffering connection with others (8 codes) 3‐ Spiritual suffering connection with nature (Code 6) 4‐ Spiritual suffering connection with God (14 codes) (Table 2).

**TABLE 2:** Defining characteristics of spiritual distress in the literature review

Number	Defining characteristics of spiritual distress	Frequency	NANDA	Not in NANDA
1	Anxiety 3,6,7,9,11^a^	5	×	
2	Cry 1,2,3,6,7,11,20^a^	7	×	
3	Expresses fatigue 3,7^a^	2	×	
4	Fear 3,6,7,8,9,11,19^a^	7	×	
5	Insomnia 3,6,7,11^a^	4	×	
6	Pain 9^a^	1		×
7	Hurt 9^a^	1		×
8	Express Lack of dignity 11,21^a^	2		×
9	Feeling frustration 9^a^	1		×
10	Feeling giving up 9^a^	1		×
11	Expresses loneliness 3,6,7,9,11,19^a^	6		×
12	Questioning identity 3,6,7,11,16,17^a^	6	×	
13	Questioning meaning of life 3,5,6,7,14,16,17^a^	7	×	
14	Questioning meaning of illness and suffering 1,2,3,7.14,18,20^a^	7	×	
15	Expresses behavioural alteration: anger 1,2,3,8,9,11,14,18,20^a^	9	×	
16	Express lac k of peace 1,2,3,4,5,6,7,10,19,20^a^	10	×	
17	Expresses lack of self‐forgiveness 8,11,12,18^a^	4		×
18	Feeling self unloved^a^		×	
19	Expresses guilt 1,2,3,11,20^a^	5	×	
20	Expresses Inadequate acceptance of what happen 5,10,12^a^	3	×	
21	Ineffective coping strategies 12^a^	1	×	
22	Expresses lack of courage 1,2,3,20^a^	4	×	
23	Perceived insufficient meaning in life^a^		×	
24	Expresses alienation 1,2,11,15,19,20^b^	6	×	
25	Refuses to interact with spiritual leader 12^b^	1	×	
26	Refuses to interact with significant other 1,2,3,7,11,20^b^	6	×	
27	Expresses Separation from support system 5,8,11,12,19,21^b^	6	×	
28	Expresses lack of creativity (singing, listening to music, writing) 3^c^	1	×	
29	Expresses lack of interest in nature 2,3^c^	2	×	
30	Expresses disinterest in reading spiritual literature 12,14^c^	2	×	
31	Expresses anger toward God/power greater than self 1,2,3,8,15,20^d^	6	×	
32	Expresses feeling abandoned by God 1,2,3,8,14,20^d^	6	×	
33	Expresses Hopelessness in relation with God/power greater than self 5^d^	1	×	
34	Inability for introspection in relation with God/power greater than self 12^d^	1	×	
35	Expresses inability to experience transcendence 2,3,16,17,19,20^d^	6	×	
36	Inability to participate in religious activities 12,13,18,19^d^	4	×	
37	Inability to pray 12^d^	1	×	
38	Perceived suffering in relation with God/power greater than self 18^d^	1	×	
39	Request for a spiritual leader 14^d^	1	×	
40	Sudden changes in spiritual practices 5,12^d^	2	×	
41	Expresses lack of meaning in life 1,2,3,7,8,15,20,21^a^	8		×
42	Expresses lack of purpose in life 1,2,8,12,20,21^a^	6		×
43	Expresses concern about beliefs and values system and/or God 2,3,5,12,13,16,17,20^d^	8		×
44	Requests spiritual support and care 1,2,3,10,20^d^	5		×
45	Express lack of hope 1,2,3,5,7,8,11,12,14,20,21^a^	11		×
46	Expresses lack of love 1,2.3,20^a^	4		×
47	Inability to express creativity 1,2,6,7,20^c^	5		×
48	Express feeling of regret 1,15,19,20^a^	4		×
49	Express feeling of temporality 2^a^	1		×
50	Express feeling of grief 2,3,10,15^a^	4		×
51	Expresses worthless 3,11^a^	2		×
52	Expresses lack of confidence 3,5^a^	2		×
53	Expresses regret and the need for forgiveness 3,5,15^a^	3		×
54	Expresses concern about the future 3^c^	1		×
55	Expresses concern about family 3,6,7,11,18^b^	5		×
56	Expresses lack of control 3^a^	1		×
57	Questions dignity 3^a^	1		×
58	Expresses feeling being lost 9^a^	1		×
59	Expresses emptiness 3,9,14^a^	3		×
60	Expresses suffering 6,7,11,12,19^a^	5		×
61	Requests to talk to a religious leader 8^d^	1		×
62	Expresses uncertainty of the future 8,1^c^	2		×
63	Expresses Lack of autonomy 11,21^a^	2		×
64	Impaired role performance 11^a^	1		×
65	Express feeling of denial 19^a^	1		×
66	Express feeling of shock 19^a^	1		×
67	Express feeling of sorrow 19^a^	1		×
68	Express feeling of discontented 19^a^	1		×
69	Express fatalism 11,18,19^d^	3		×
70	Expresses Giving up the life 19^a^	1		×
71	Express feeling of pessimistic 19^a^	1		×
72	Expresses dissatisfaction with others 19^b^	1		×
73	Expresses disobedience to others 19^b^	1		×
74	Expresses not forgiving others 19^b^	1		×

^a^ Related to Spiritual suffering connection to self.

^b^ Related to Spiritual suffering connection with others.

^c^ Related to spiritual suffering connection with nature.

^d^ Related to spiritual suffering connection with God.

This classification was based on the definition provided by the NANDA‐I for the diagnosis of spiritual distress nursing. The obtained codes were most frequently associated with the sub‐category of spiritual suffering. The high number of codes (48 codes) were related to the category of spiritual suffering concerning the self. Articles with codes 1‐3, 7, 11, 19 and 20 had the largest share (15–30 codes) of the extracted codes. The article with code 3 with 33 codes extracted had the highest amount. Based on the results of this combined review, the criteria with the highest frequency and repetition in articles were lack of calm, hope, change in anger behaviour, meaning in life, change in fear and crying behaviour, a disorder in the system of values and beliefs. After comparing these criteria with the criteria provided by Nanda, it was found that only 33 of them were mentioned as defining characteristics of spiritual distress in the classification of NANDA‐I classification for nursing diagnoses (Table 2).

## 4 DISCUSSION

The purpose of this review was to determine the specifications of spiritual distress in related literature to validate the defining characteristics attributed to it as a nursing diagnosis. Finally, 74 specifications were extracted, which according to the NANDA‐I taxonomy (2018–2020) definition of spiritual distress, were categorized as distress concerning self, others, nature, or power greater than self. Such a classification helps the more accurately diagnose spiritual distress. The high accuracy of diagnostic features of a nursing diagnosis makes the care plan designed for the client effectively in any particular area.

This categorization has been removed in the NANDA‐I taxonomy version 2021–2023 (Ackley et al., 2021). This study was conducted before the latest version of the NANDA‐I taxonomy 2021–2023, and we followed the previous format of listing the defining characteristics of spiritual distress. Many of the defining characteristics of spiritual distress in the recent version still need further studies to increase accuracy and precision due to the overlap with the defining characteristics of other diagnoses so that clinical nurses can accurately diagnose spiritual distress through them. For example, nursing diagnoses of anxiety and frustration have overlapped in defining characteristics and related factors to spiritual distress (Herdman & Kamitsuru, 2017).

It seems that for a higher differentiation ability, it is necessary to present defining characteristics of spiritual distress based on the cultural and religious background of the individual. The defining characteristics of spiritual distress also mentioned in other nursing diagnoses should be replaced with more specific terms.

Consistent with the nature of an integrative review, this review included studies with various methodologies. Such a variety of methods of investigation shows the complexity and multidimensional subject of spiritual distress. The triangulation methods of study are suggested when the aim is to investigate the different aspects of a complicated construct(Creason, 2004).

One of the considerations was a large number of validation studies for the diagnosis of spiritual distress compared to other nursing diagnoses. It can be attributed as an attempt to reduce the ambiguity of defining features and increase their ability to differentiate.

Nurses are the first group of health professionals to be in contact with people who face spiritual distress. Therefore, they need preparation to respond appropriately in such situations (Narayanasamy, 2006). The results of validation articles will help nurses accurately diagnose spiritual distress in different cultures to provide or promote spiritual care.

The authors of most of the reviewed articles were from the discipline of nursing. It can be concluded that nurses are pioneers in the field of spirituality and related concepts. The definition of nursing provided by the International Nursing Association emphasizes the importance of adopting a holistic and patient‐centred approach to nursing care, which is at the heart of the American Holistic Nursing Association's mission statement (Helming et al., 2013). Based on the definition of nursing defined by the Royal School of Nursing, the nurse notes the person as a whole and their responses to health problems, illnesses, and disabilities, including their spiritual responses. The Royal College of Nursing identified spiritual support as the essential part of the nursing role (Daly et al., 2019). On the one hand, spirituality is considered one of the four dimensions of a person's health and well‐being. Nursing is a holistic discipline that pays attention to all physical, mental, psychological, and spiritual dimensions of health. For this reason, nurses and other clinical members of the health care team need to identity, diagnose and support clients with spiritual distress as part of providing holistic care (Caldeira et al., 2017b). Due to this felt need in nursing, nurses as the primary caregivers have paid more attention to the field of spirituality (Caldeira et al., 2013).

The subject of most studies in this review was cancer, which indicates that the experience of cancer tremendously affects a person's sense of spirituality. Cancer is often known as a life‐threatening disease, and its diagnosis causes meaning‐seeking and purpose‐seeking behaviours in the affected patients. Many cancer patients have unmet spiritual needs (Caldeira et al., 2017b). Spiritual distress is associated with increased pain, depression, and suffering in cancer patients and should be identified and treated in a healthcare setting to improve such symptoms (Caldeira et al., 2016).

This review extracted the most frequent defining characteristics in the resources. Lack of peace, a sense of hopelessness, changes in anger behaviour, changes in the meaning given to life, fear, frequent crying, and disturbance in the system of beliefs were of them.

The lack of peace (Caldeira et al., 2013; Caldeira et al., 2014; Caldeira et al., 2016; Caldeira et al., 2017b; Chaves, Carvalho, & Hass, 2010; Chaves, Carvalho, Terra, & Souza, 2010; Hatamipour et al., 2015; Ku et al., 2010; Schultz et al., 2018; Simão et al., 2015) and lack of hope (Caldeira et al., 2013; Caldeira et al., 2017a; Chaves, Carvalho, & Hass, 2010; Chaves, Carvalho, Terra, & Souza, 2010; Glenn & Pieper, 2019; Hatamipour et al., 2015; Martins & Caldeira, 2018; Pinho et al., 2017; Simão et al., 2015; Yang et al., 2012) were the most common defining characteristics mentioned in almost half of the reviewed studies. Both of these characteristics were related to the subcategory of spiritual suffering concerning the self. In the study of Caldeira et al, it was concluded that lack of peace and hope were the most common defining characteristics of spiritual distress(Caldeira et al., 2013). Hatamipour et al., in their qualitative study, aimed at determining the spiritual needs of cancer patients, named peace‐seeking as one of the main themes. In this study, hope was the subcategory of the peace‐seeking theme (Hatamipour et al., 2015). Lopez Chaves et al., in their study, stated that lack of peace (Chaves, Carvalho, & Hass, 2010; Chaves, Carvalho, Terra, & Souza, 2010) and lack of hope (Chaves, Carvalho, Terra, & Souza, 2010) are the most important defining characteristics with a high frequency. According to Caldeira, lack of peace is a major defining characteristic of nursing diagnosis spiritual distress (Caldeira et al., 2016). Schultz et al. reported the lack of inner peace as one of the main statements of spiritual distress (Schultz et al., 2018).

In their study, Velosa et al. reported that patients with spiritual distress as a nursing diagnosis have more than one defining characteristic; and anger behaviour was one of the most important characteristics (Velosa et al., 2017). In a clinical validation study on impaired spirituality, anger behaviour was the most common characteristic (Chaves, Carvalho, Terra, & Souza, 2010).

Pinho et al., reported a lack of meaning in life and asking the meaning of life, along with the lack of purpose, as the major defining characteristics of spiritual distress (Pinho et al., 2017). In Chaves's study, the lack of meaning in life ranked third among the other seven most important defining characteristics of spiritual distress (Chaves, Carvalho, & Hass, 2010). They reported the lack of meaning in life as one of the defining characteristics of spiritual distress in patients with chronic diseases (Chaves, Carvalho, Terra, & Souza, 2010). Hatamipour et al., in their qualitative study based on interviews with cancer patients, extracted this characteristic as one of the main themes (Hatamipour et al., 2015). Caldeira et al., reported asking the meaning of life as one of the major defining characteristics of spiritual distress (Caldeira et al., 2016; Caldeira et al., 2017a). But in the other study, the lake of meaning of life was reported as a minor defining characteristic of spiritual distress (Caldeira et al., 2017a).

In the studies conducted by Caldeira et al., behaviour change, fear, and crying, along with ten other characteristics, were reported as major criteria for spiritual distress (Caldeira et al., 2016; Caldeira et al., 2017a). Also, they mentioned these two criteria as defining characteristics of spiritual distress in their integrated review, these two criteria were mentioned along with other criteria as defining characteristics of spiritual distress (Caldeira et al., 2013).

Concern about the belief system or God has been reported in several studies as a defining characteristic of spiritual distress (Caldeira et al., 2013; Glenn & Pieper, 2019; Monod et al., 2010; Monod et al., 2012; Simão et al., 2015). In the qualitative research of Hatamipour et al., strengthening spiritual beliefs, communication with God, and prayer, as subcategories of the theme of transcendence, were from the spiritual needs of cancer patients (Hatamipour et al., 2015). In the study of Chaves et al., concern about the belief system and/or God was reported as the first and most important criterion among the seven main defining characteristics of the nursing diagnosis of spiritual distress. They reported this characteristic with a frequency of 27.3% has little correlation with the nursing diagnosis of spiritual distress. However, the researchers in this study stated that they extracted this criterion, along with several other characteristics, through an integrated review of literature, as the first step of their research (Chaves, Carvalho, Terra, & Souza, 2010). Caldeira et al., reported similar results and concluded that this characteristic was irrelevant to the nursing diagnosis of spiritual distress (Caldeira et al., 2017a). This criterion was common in our integrated review study. On the other hand, other studies have reported conflicting results about this criterion as defining characteristic of the nursing diagnosis of spiritual distress. Therefore, it is necessary to conduct other quantitative and qualitative studies taking into account the views of patients and experts in the field of spirituality to identify the phenomenon of spiritual distress as much as possible and provide specific high‐quality care for it. It should be noted that this contradiction can be due to different samples or cultural and religious backgrounds of the people participating in the studies.

Most of the extracted codes were related to spiritual distress toward self means spirituality, and related concepts such as spiritual distress are phenomena based on the individual, internal and subjective. Most of the researchers were seeking to discover individual dimensions of the concept of spiritual distress. As a result, nurses can better differentiate between the symptoms of spiritual distress and the characteristics of similar nursing diagnoses if the angles of this concept are identified with the individual. In this way, nurses in clinical settings can more effectively apply this diagnosis to their patients, provide appropriate care, and experience a greater sense of confidence. These factors increase patients' satisfaction from nurses and nurses' satisfaction from themselves.

The defining characteristics extracted in this study propose 41 more criteria that The NANDA‐I classification does not mention in the list of defining characteristics of nursing diagnosis of spiritual distress. Nurse researchers need to consider these proposed criteria in the literature. Nurses should evaluate and clinically validate them; to add the more sensitive and specific ones for diagnosing spiritual distress.

### 4.1 Limitations

Combination of articles with different methodology, design and research question was complex and challenging. Nevertheless, in this integrative review using the systematic approach and rigorous methodology by Broom, and explicit methods for data analysis was very helpful (Broome & Company, 2000). In addition, screening and quality evaluation of the included articles in the study was performed by two authors.

## 5 CONCLUSION

According to the findings of this study, lack of peace, hopelessness, anger, change in the meaning given to life, fear, and crying were the most defining features in the reviewed texts for the diagnosis of spiritual distress nursing.

As a result, these defining characteristics can lead clinicians to consider the nursing diagnosis of spiritual distress for their patients. Determination of the defining characteristics that most likely present a nursing diagnosis facilitates the provision of appropriate and individualized care. The emphasized view of holistic care in nursing necessitates attention to the spiritual dimension of care as much attention to spiritual care as it does to other aspects of human health.

## AUTHOR CONTRIBUTIONS

FE, LN, and AZ: Study design. FE, LN, AZ: Data collection, data analysis and interpretation. FE, LN: Manuscript writing. FE, LN, AZ: Critical revisions for important intellectual content.

## CONFLICT OF INTEREST

We have no conflict of interest to declare.

## FUNDING INFORMATION

This study was supported by Shahid Beheshti University of Medical Sciences. The funder had no role in the design and writing the manuscript and will have not in data collection and analysis of data.

## ETHICAL APPROVAL

As a part of doctoral dissertation, this study has been approved by the ethics committee of Shahid Beheshti University of Medical Sciences. (IR.SBMU.PHARMACY.REC.1398.291).

## PATIENT CONSENT

Patient consent statement was not required.

## Data Availability

The data that support the findings of this study are available from the corresponding author upon reasonable request.
